# FDF-PAGE: a powerful technique revealing previously undetected small RNAs sequestered by complementary transcripts

**DOI:** 10.1093/nar/gkv604

**Published:** 2015-06-13

**Authors:** C. Jake Harris, Attila Molnar, Sebastian Y. Müller, David C. Baulcombe

**Affiliations:** 1 Plant Sciences Department, Cambridge University, Cambridge, CB2 3EA, UK; 2 School of Biological Sciences, Edinburgh University, Edinburgh, EH9 3JH, UK

## Abstract

Small RNAs, between 18nt and 30nt in length, are a diverse class of non-coding RNAs that mediate a range of cellular processes, from gene regulation to pathogen defense. They guide ribonucleoprotein complexes to their target nucleic acids by Watson–Crick base pairing. We report here that current techniques for small RNA detection and library generation are biased by formation of RNA duplexes. To address this problem, we established FDF-PAGE (fully-denaturing formaldehyde polyacrylamide gel electrophoresis) to prevent annealing of sRNAs to their complement. By applying FDF-PAGE, we provide evidence that both strands of viral small RNA are present in near equimolar ratios, indicating that the predominant precursor is a long double-stranded RNA. Comparing non-denaturing conditions to FDF-PAGE uncovered extensive sequestration of miRNAs in model organisms and allowed us to identify candidate small RNAs under the control of competing endogenous RNAs (ceRNAs). By revealing the full repertoire of small RNAs, we can begin to create a better understanding of small RNA mediated interactions.

## INTRODUCTION

Genome regulation by small RNAs (sRNA) is a feature of all eukaryotes, including plants, animals and fungi. There are three main types of small RNAs: (i) microRNAs (miRNAs), which target genes and have important roles in development and in stress-responses (1–3), (ii) small interfering RNAs (siRNAs), which target transposons and repetitive elements to mediate genome integrity (4–6) and (iii) virus-derived small RNAs, which are involved in antiviral defense (7–12). The core mechanism shared by these pathways and across species involves the recognition of double stranded RNA by an RNase III domain containing Dicer enzyme, triggering cleavage into short 18–30 bp small RNA duplexes. Individual sRNA strands can then be loaded into Argonaute proteins to guide sequence-specific targeting of complementary nucleic acids.

sRNAs exert control over the genome at the transcriptional, post-transcriptional and translational levels, yet the mechanisms regulating the sRNAs themselves remain poorly understood. Recently, a regulatory mechanism was discovered (13) in which miRNAs are sequestered or ‘sponged’ by competing endogenous coding or non-coding RNAs (ceRNAs), (14–17) that may be linear or circular, (18,19). ceRNAs not only provide a mechanism for transcripts with shared miRNA binding sites to sense and control one another's abundance, but their presence suggests that the cellular availability of miRNAs is limited and tightly regulated.

To investigate the diverse cellular functions of sRNAs, it is essential to gain an accurate profile of sRNA abundance in the cell. However, researchers studying sRNAs typically begin by extracting total ribonucleic acid from a tissue sample, resulting in a sample that contains both the sRNAs of interest and their complementary transcripts. It is possible that hybridization to these complementary RNAs could interfere with small RNA detection. Indeed, addition of synthetic RNA as a miRNA inhibitor can affect the detection of complementary miRNAs (20) and a more recent study suggested that genomic viral RNAs could mask the detection of complementary virus-derived sRNAs (21).

To fully understand the effect of complementary RNAs on sRNA detection, we mixed synthetic RNAs *in vitro*. We show here that complementary RNA reduced the small RNA signal by northern blotting in as little as 10-fold molar excess and reduced sRNA representation in sequencing libraries. To overcome this problem we developed FDF-PAGE, which results in full denaturation and signal recovery even in the presence of 1000X fold excess complementary RNA. FDF-PAGE therefore provides a more robust representation of the sRNA profile than previous methods and will be a useful technique for routine analysis of sRNA. In addition, by comparing sRNA cloning and high-throughput sequencing from FDF-PAGE and non-denaturing methods we were able identify known and novel candidate sRNAs that are under the control of ceRNAs in *Arabidopsis thaliana*, *Mus musculus*, *Drosophila melanogaster* and *Caenorhabditis elegans*. These results demonstrate the importance of full denaturation during the analysis of endogenous sRNA profiles.

## MATERIALS AND METHODS

### RNA pre-loading treatments

**F:** RNA was mixed in a 1:1 ratio with 2× FDE (de-ionised formamide, 0.5 mM EDTA, pH 8.0 + dyes (0.1% w/v bromophenol blue + 0.1% w/v xyelene cyanol)), incubated at 65°C for 5–10 min then snap cooled on ice before loading (22 – using TBE method). **100% formamide:** RNA was dessicated then re-suspended in 2× FDE, incubated at 98°C for 5 min and loaded directly (21). **FDF:** RNA in 4 ul volume was mixed with 11 ul of FDF buffer (2.75 ul of formaldehyde (40%), 7.5 ul of de-ionized formamide, 0.75 ul of 10× MOPS buffer [200 mM MOPS, 50 mM sodium acetate, 10 mM EDTA, pH 7.0]) and incubated at 55°C for 15 min. 2 ul of dyes was added at room temperature before loading (23). *Note*: FDF treated samples MUST be run in MOPS-based gels and running buffer (see FDF-PAGE protocol in supplemental materials). **Glyoxal/DMSO:** RNA in 4 ul volume was mixed with 1.5 ul of TBE (90 mM Tris-borate, 2 mM EDTA), 7.5 ul of DMSO and 2 ul of de-ionised glyoxal. Samples were incubated at 50 for 1 h and snap cooled on ice. 2 ul of dyes was added before loading (23). **8.5M urea:** RNA in 4 ul volume was mixed with 8.5M urea buffer (8.5M urea, 2× TBE + dyes) and boiled at 98°C for 5 min before loading.

#### Denaturing PAGE

Pre-loading treated RNA samples were loaded in to 0.75 mm polyacrylamide–urea gels (7M urea, 15% 19:1 acrylamide:bis-acrylamide, 0.5× TBE (or 0.5× MOPS buffer if using FDF treated RNA), 700 mg/l ammonium persulphate and 350 ul/l tetramethylethylenediamine). Gels were run in a 0.5× TBE (or 0.5× MOPS buffer if using FDF treated RNA) running buffer at up to 150 V until the bromophenol blue reached the bottom of the gel.

#### Size selection

After visualization of nucleic acid by SYBR Gold (Life Technologies) staining on a dark reader, polyacrylamide slices containing the small RNA band were excised and placed into a shredder (0.5 ml microcentrifuge tube with three 21 G needle holes punctured into the bottom within a 2 ml nuclease free microcentrifuge tube) and spun at 10 000 × g. Three volumes of 0.3 M NaCl (pH 7.0) was added per volume of gel and rotated at 4°C overnight. NaCl + gel was transferred into a SpinX column (Costar) and spun at 17 000 × g for 2 min at 4°C. Flow through was transferred to a nuclease free siliconised 1.5 ml tube and ethanol precipitated, including two washes of the pellet in 80% ethanol. RNA was re-suspended in nuclease free water.

#### Northern blots

were performed as described by (22 – TBE method) with the modifications indicated described above. Decade marker (Ambion) was used as a ladder.

#### Gel shift assay

21_S sRNA oligonucleotide was 5′ end labeled with γ-^32^P-ATP prior to mixing with RNA (as indicated) and separated by denaturing PAGE. The gel was exposed directly to a phosophor imager plate and then was stained by SYBRgold for total nucleic acid visualization.

#### sRNA qPCR

For small RNA qPCR the 21_S sRNA oligonucleotide was first 5′ phosphorylated using PNK forward reaction in the presence of ATP. The phosphorylated oligonucleotide was mixed with 1ug of *Arabidopsis* total RNA and competitor oligonucleotide (as indicated) were subjected to the illumina small RNA cloning protocol up to and including cDNA synthesis. qPCR was performed with the primers indicated using SYBR green (Thermo Scientific) mastermix.

#### Small RNA libraries

5–10 ug of total RNA was used as the starting material for all small RNA libraries. All libraries were generated and sequenced according to the Illumina TruSeq small RNA cloning protocol (RS-200-9002DOC) with any modifications indicated.

#### RNA samples

Total RNA from *A. thaliana* Col-0 floral tissue, mixed sex adult *D. melanogaster* Oregon-R strain, *C. elegans* N2 strain and *M. musculus* testis 25 days post-partum (25 dpp) were extracted by TRIzol reagent (Invitrogen). CymRSV infected *Nicotiana benthamiana* total nucleic acid was extracted by standard protocol based on (24). HPLC purified RNA oligonucleotides were ordered from MWG. 40_Shuff was selected by randomly shuffling the 40_AS oligonucleotide sequence until a similarly low secondary structure (both at Δ*G* = −1.9, predicted by mFold) was identified.

#### Bioinformatics

sRNA reads were trimmed and size selected (between 15nt and 30nt) and aligned by PatMaN without mismatches to the respective genomes. Multi-mapping reads files were selected for downstream analysis. Reads were normalized with the TMM method as implemented by the ‘segmentSeq’ package (BioConductor). Differential expression analysis was performed using baySeq. Genomes: Cymbidium ringspot virus (GenBank accession NC_003532.1); *A. thalinana* (genome and annotation) from The Arabidopsis Information Resource (TAIR10); *N. benthamiana* from the Sol Genomics Network (v0.42); *M. musculus* annotation and genome (mm9) from ensemble.org and transposon annotation from hgdownload.cse.ucsc.edu; *D. melanogaster* from ftp.flybase.net (Release 5.51); *C. elegans* genome and annotation for version WS236 from ftp.wormbase.org. Drosophila piRNAs datasets were obtained from piRNABank (http://pirnabank.ibab.ac.in/) and Mouse piRNAs from piRBase as of 14.8.2013 (http://regulatoryrna.org). miRNAs were identified by matching against mature miRNAs from miRBase release 20. piRNAs were identified in the same manner, cross matching sRNAs to the respective piRNA resources. MA Plots: Normalized reads (library size estimate TMM method) from FDFSS and NSS libraries were analyses; y-axis (M) shows log_2_ fold change [log_2_(FDFSS/NSS)] and x-axis (A) shows average abundance [$\frac{1}{2}$log_2_(FDFSS*NSS)]. Bioinformatic scripts are available on request from S.Y.M. Libraries are available for download at arrayexpress E-MTAB-2733.

## RESULTS AND DISCUSSION

### Complementary RNA affects small RNA detection

**Figure 1.**
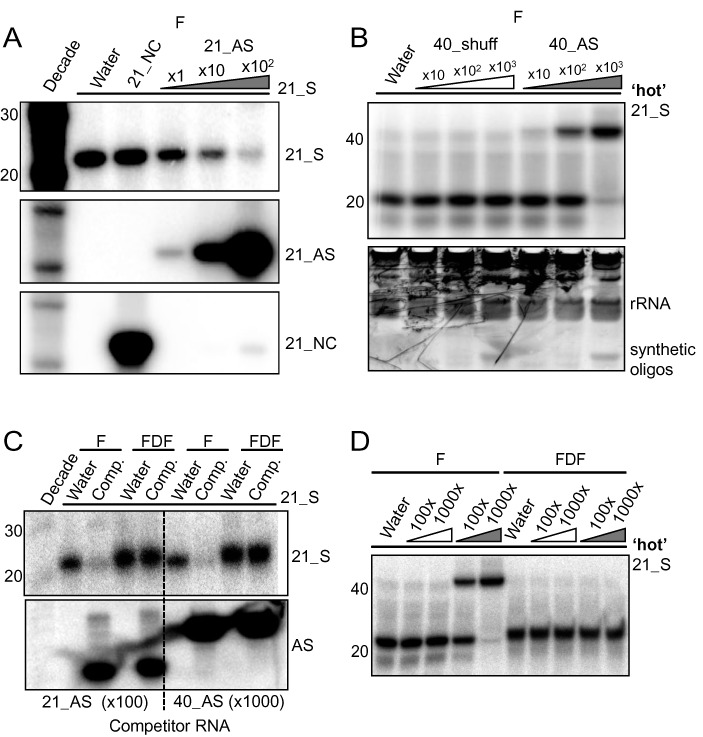
FDF-PAGE promotes full denaturation of small RNA duplexes. (**A**) Increasing concentrations of antisense 21nt oligonucleotide reduces small RNA signal by northern. Northern blot with a 21nt sense (21_S) RNA oligonucleotide at 2 nM mixed with equimolar, ×10 or ×100 concentration of 21nt antisense (21_AS) RNA oligos. Water and 21_NC (a non-complementary 21nt RNA oligonucleotide at x100 molar excess) are used as negative and sequence specificity controls, respectively. Decade marker is used as a ladder. Middle panel; membrane is stripped and re-probed for loading of 21_AS. Lower panel; membrane is stripped and re-probed for loading of NC_21. ‘F’ indicates traditional formamide denaturing PAGE treatment. (**B**) Gel shift assay. 5′ end radiolabeled (γ ^32^P, ‘hot’) 21_S RNA (2nM) is mixed with 4 ug *A. thaliana* total RNA and equimolar, x100 or x1000 molar excess 40nt oligonucleotides antisense (40_AS) or randomly shuffled (40_Shuff), using F pre-loading treatment, separated by denaturing PAGE and exposed to a phosphor imager plate. Lower panel, radioactive gel was stained by SYBR Gold then wrapped in protective cling film before taking a UV image to visualise RNA loading. (**C**) Northern blot showing FDF treatment releases sequestration of 21_S (2 nM) in the presence of 100x molar excess 21_AS or 1000x molar excess 40_AS RNA oligonucleotides. Lower panel, membrane is stripped and re-probed for loading of competitor RNA. (**D**) Gel shift assay. Radiolabeled (γ-^32^P, ‘hot’) 21_S RNA (2 nM) is mixed with 40_Shuff (white triangle) or 40_AS (gray triangle) at x100 and x1000 molar concentrations and subjected to F or FDF treatments prior to denaturing PAGE.

Northern hybridization is a standard and widely used approach to identify, validate and quantify the levels of sRNAs in plant and animal extracts (25,26). Current techniques involve denaturation of the RNA in formamide (F) prior to electrophoresis through a 7M urea polyacrylamide gel (F-PAGE, so called ‘denaturing’ PAGE)(22). To determine whether detection by northern hybridization could be influenced by the presence of complementary RNA we mixed a synthetic 21nt sRNA at physiological concentrations, 2 nM, (27,28) with 21nt or 40nt RNA oligonucleotides prior to electrophoresis and blotting. From these assays, we could see that the presence of complementary oligonucleotides in molar excess caused a dose-dependent reduction in the hybridization signal (Figure 1A, Supplemental Figure S1A).

In a gel shift assay, in which the sRNA was radiolabeled prior to denaturing PAGE, the sRNA was physically sequestered rather than degraded by the complementary 40mer oligonucleotide in the presence of 4 ug of total RNA from *A. thaliana* (Figure 1B). These results demonstrate that sRNA duplexes remain stable in 7M urea gels and, as the concentrations of sRNA and complementary RNA are were within the range found within cells (27), the standard northern assay may not give an accurate estimate of sRNA abundance.

Next, we turned our attention to sRNA cloning, as it is the preferred method for analysis of global sRNA profiles (9–10,29–30). The recently developed Illumina sRNA library preparation protocols allow direct cloning from total RNA extracts but the presence of complementary RNA might introduce an sRNA cloning bias (31). To test this hypothesis, we mixed 1μg of *A. thaliana* total RNA with a synthetic sRNA at physiological concentrations (28) and a 1000-fold molar excess of complementary or randomized 40nt RNA competitor. We then proceeded with the Illumina sRNA cloning protocol (3′ and 5′ adapter ligation, cDNA synthesis) and used a modified qPCR to quantify the cloned synthetic sRNAs. The data revealed a 6.5–18.5 reduction in representative abundance of the synthetic sRNA in the presence of the 40nt complementary RNA (Supplemental Figure S2). We conclude that the adapter ligases and/or the cDNA synthesis primers in the sRNA cloning protocol have a preference for single stranded RNA over sRNA in a duplex so that sRNA representation in the library is reduced if base-paired duplex structures are formed *in vivo* and/or after RNA isolation.

### FDF-PAGE treatment dramatically increases denaturation efficiency

To overcome the potential for sRNA sequestration we tested several treatments including boiling the RNA in 100% formamide (21), 8.5M urea (http://bartellab.wi.mit.edu/protocols.html), glyoxal/DMSO and formaldehyde treatment (23). We reasoned that, if the RNA could be denatured prior to loading, it would remain denatured as it migrated through the 7M urea gel. Of these treatments, only formaldehyde (hereafter referred to as fully denaturing formaldehyde polyacrylamide gel electrophoresis, FDF-PAGE) abolished sequestration completely - returning full signal by northern and releasing hybridized sRNAs during gel shift, in the presence of x100 (21nt) or x1000 (40nt) fold molar excess complementary RNA (Figure 1CD, Supplemental Figure S1BC).

The effectiveness of FDF-PAGE is likely due to two factors. First is that, as designed, it prevents sequestration of sRNAs by their complement. Second is because the sRNA is only exposed to formaldehyde in the treatment before electrophoresis. By minimizing prolonged exposure to formaldehyde we reduce the likelihood of stable modification of the amino groups in the bases (32). Other denaturing methods of denaturing RNA gel electrophoresis involve prolonged exposure to formaldehyde because it is included in the electrophoresis buffer. An additional modification to the protocol that we have not tried might involve combining FDF-PAGE with carbodiimide-crosslinking to further enhance sensitivity of small RNA detection by northern blotting (33).

### Viral small RNAs are derived equally from both strands of the viral genome

**Figure 2.**
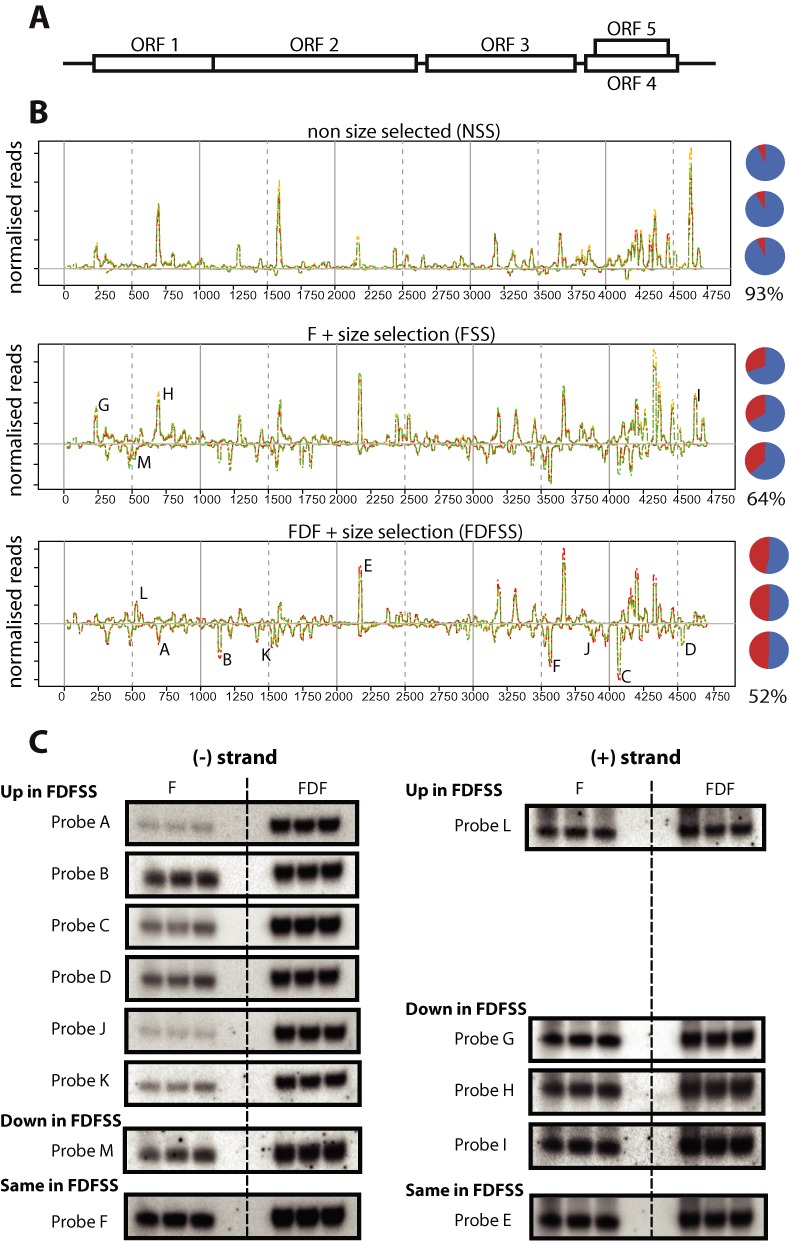
CymRSV vsRNA strand bias is abolished under denaturing FDFSS conditions. (**A**) Schematic of CymRSV genome. (**B**) Distribution of vsRNA along the CymRSV genome. Above the line are (+) strand, below the line are (−) strand in three independent biological samples (red, green and yellow lines) under three different treatments, non size selected (NSS), F-PAGE + size selected (FSS), or FDF-PAGE + size selected (FDFSS). Pie charts show the proportion of (+) (blue) or (−) (red) vsRNA mapping reads for each library with average (+) strand percentage shown beneath. (**C**) Northern blot for hotspots using the same CymRSV RNA used to make the libraries shown in (B). (−) Strand hotspots that are enriched under FDFSS were validated using probes A, B, C, D, J and K. Hotspots that were reduced under FDFSS (probes G, H, I and M) were not reduced or even increased under FDF-PAGE. Increased denaturation should never result in reduced signal by northern blot. This suggests that the reduced representation of G, H, I and M hotspots under FDFSS (as compared to FSS) reflects their true relative abundance in the small RNA pool once all the sRNAs have been released. Probes E and F serve as loading controls, probing for hotspots that are equally represented in FSS and FDFSS libraries on the (+) and (−) strands, respectively.

Having shown that the presence of complementary RNA can affect the detection of sRNAs by northern blotting and sRNA cloning, we tested the possibility that sRNA sequestration could pose a problem in biological samples. Many viruses encode positive-sense single-stranded RNA (ssRNA) genomes that are targeted by the host antiviral RNA silencing machinery, generating vast quantities of viral derived small RNAs (vsRNAs) (7). These vsRNAs are often reported to have a positive sense strand bias (34–37). A recent report suggested that genomic viral RNA might sequester complementary vsRNAs during gel electrophoresis (21). We chose to investigate CymRSV, a positive sense ssRNA plant virus, for which >90% of the vsRNA sequences are reported to derive from the plus strand (34–36). We generated sRNA libraries from three individual CymRSV infected *N. benthamiana* plants using the standard Illumina protocol from RNA that was non-size selected (NSS) and from samples subjected to F-PAGE or FDF-PAGE and size-selection treatments (FSS and FDFSS, respectively) (Figure 2).

In agreement with the previous reports, the NSS sRNA libraries showed >90% strand-bias towards the plus strand vsRNAs. However, the FSS treated vsRNAs showed a reduced strand bias of ∼65%, while vsRNAs from the fully denaturing FDFSS treated small RNA libraries mapped *equally* to both strands. This marked difference in strand ratio between treatments indicates that the increased strength of denaturation results in decreased artifactual bias to positive strand vsRNAs. The likely explanation of these data is that the highly abundant (+) sense viral RNA genome (38) binds and sequesters (-) sense vsRNAs (21) but that, under fully denaturing conditions, these vsRNAs are released from viral RNA:vsRNA hybrid.

The reported and consistently repeatable positive strand bias in vsRNAs led to the suggestion that vsRNAs are predominantly generated from local foldback structures in the (+) viral genome (35,36). However, the equal amount of (+) and (−) strand specific vsRNAs in the FDFSS libraries supports the hypothesis that vsRNAs are generated from long double-stranded RNA precursors. This latter interpretation is consistent with the observed requirement for double-stranded RNA generating RNA-dependent RNA polymerases (RDRs) in antiviral defense (39–42).

The distribution of vsRNAs along the length of the CymRSV genome was non-uniform (Figure 2B). These vsRNA ‘hotspots’ could reflect sRNAs that are stabilised or thermodynamically favored by Argonautes (43,44) or they could reflect an artifact caused by small RNA cloning bias (31). We used northern blotting from FDF-PAGE to verify six (−) sense vsRNA that were enriched in sequence libraries under the FDFSS treatment, (Figure 2BC; probes A, B, C, D, J and K). The hotspots on the (+) and (−) strands that were reduced in the FDFSS sequence libraries were not affected in northern analysis by FDF-PAGE (e.g. Figure 2BC probes G, H, I and M), suggesting their reduced representation in FDFSS reflects their true relative abundance in the small RNA pool once all the sRNAs have been released. From these data we conclude that the hotspots observed under FDFSS likely resemble the state *in vivo*. An illustration of how sequestration has affected other datasets is with CymRSV (36), for which the small RNA data were topologically similar to our non-size selected libraries (Supplemental Figure S3D).

### Endogenous *N. benthamiana* small RNAs are affected by FDFSS treatment

The small RNAs that map to the *N. benthamiana* genome were also affected by FDFSS treatment. As expected, there were predominant 21nt and 24nt small RNA size classes irrespective of the method used (Supplemental Figure S4). However, the 21nt sRNAs were relatively more abundant in the FDFSS treated samples. The proportion of miRNAs was roughly ten fold higher in FDFSS than NSS samples, with the FSS samples being intermediate. This finding suggests that miRNAs may be sequestered by complementary endogenous RNAs in the NSS samples.

### Identifying candidate small RNAs under the control of ceRNAs

**Figure 3.**
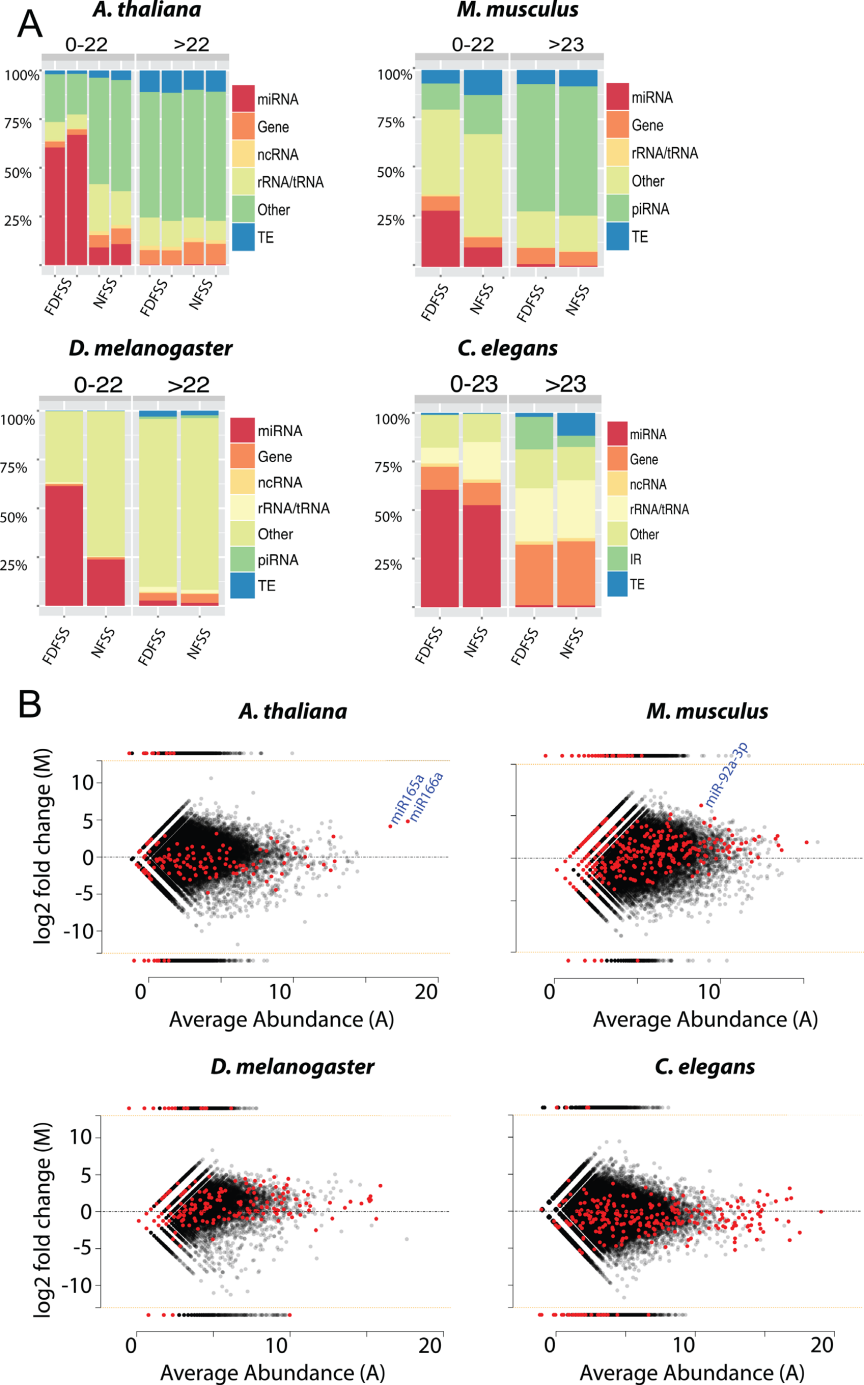
miRNA sequestration revealed by comparison of FDFSS versus NSS treatments. (**A**) Proportion of sRNAs mapping to different genome annotation features (miRNA = miRNA, Gene = gene, ncRNA = non-coding RNA, rRNA/tRNA = ribosomal/transfer RNA, Other = non-annotation mapping, TE = transposable element, piRNA = piRNA, IR = inverted repeat) in the four eukaryotes. sRNAs are split by size class (indicated on top) illustrating size specific effects. (**B**) Comparison of small RNA representation in FDFSS versus NSS libraries using MA plot. Each point represents a small RNA species. Points above the line are enriched in FDFSS libraries (y-axis = log_2_ fold change FDFSS/NSS). X-axis shows small RNA average abundance. miRNAs are indicated in red, non-miRNA species are in gray. miR166/165 in *A. thaliana* and miR-92a-3p in *M. musculus* (indicated in blue) are highly FDFSS enriched miRNAs that have previously been identified as under the control of ceRNAs.

ceRNAs could account for under-representation of miRNAs in the NSS samples. To identify candidate miRNAs under ceRNA control we prepared sRNA libraries from *A. thaliana* (floral), *D. melanogaster* (whole), *C. elegans* (whole) and *M. musculus* (testis, 25 dpp). The samples were prepared directly from total RNA (NSS) or after FDF-PAGE treatment and size selection (FDFSS) as these treatments have the largest difference in denaturation efficiency (see Figure 2) and would be most sensitive to sequestration. There was a higher proportion of miRNA mapping reads in all four organisms under the denaturing FDFSS treatment, while other sRNA classes, for instance the piRNAs in mouse testis, remained largely unaffected (Figure 3, Supplemental Figure S5). These results are consistent with the possibility that ceRNA sequestration of miRNA but not other sRNAs may be widespread in these organisms.

In *A. thaliana*, a drastic increase in miRNA representation (from ∼10% to ∼60% in of the total small RNA library and an overall predominance 21nt over 24nt sRNAs) in the FDFSS libraries could be largely (but not entirely, see Supplemental Figure S6) attributed to a 25-fold enrichment of the highly expressed miR166 and miR165 (Figure 3A). MiR166/165 target HD-ZIP III transcription factors during development (45,46) and have previously been identified as targets for sequestration by a non-coding endogenous transcript containing a non-cleavable miR166 binding site (47). miR166/165 acts as a mobile morphogen (48) and it is possible that its native mRNA targets and miRNA target mimics may titrate and fine tune its activity in different tissues.

Similarly, the most highly enriched miRNA in the mouse samples, miR-92a (∼70 fold higher in FDFSS than NSS, Figure 3B), along with a number of other FDFSS enriched miRNAs (including the let-7 family, miR-25, miR-26a and miR-181) have already been described as under regulation by competing endogenous transcripts important for the control of cancer (17,49–50).

These findings with *Arabidopsis* and mouse validate the comparison of FDFSS versus NSS to identify biologically relevant sequestered sRNAs and suggest that the comparative data from these libraries will provide a valuable resource (see Supplementary Data). However, some small RNAs may only be sequestered *in vivo* when in complex with Ago and therefore might not be identified using this approach. miRNAs under sequestration have not yet been described for *D. melanogaster* or *C. elegans* but our results indicate that miR-956 and miR-90, respectively, might be good candidates to investigate in these organisms (Supplementary Data).

We wondered whether miRNAs that were released from the long RNA pool might sequester their cognate miRNA* strands and reduce their cloning efficiency. To investigate this possibility we picked the top five most significantly FDFSS enriched miRNAs from each organism and determined the representation of their miRNA* strands under FDFSS or NSS. There were no consistent patterns of enrichment, with some miRNA* increasing under FDFSS and others decreasing (Supplemental Figure S7). It is likely therefore that miRNAs do not re-associate with their star strand after FDFSS or, if they do, that the 2nt 3′ overhangs from the dicer cleavage may be sufficient to allow ligation of the 3′ adapter.

### FDFSS treatment does not alter sRNA sequence composition

**Figure 4.**
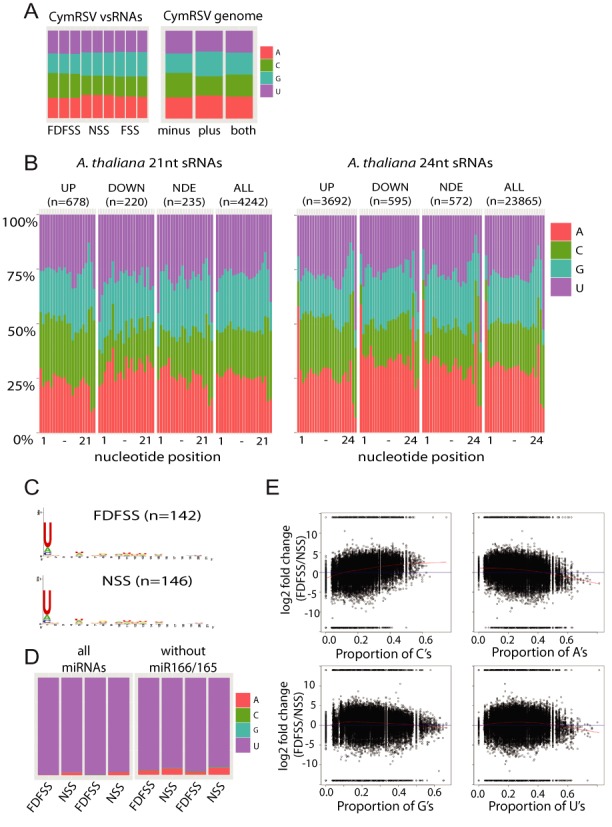
FDFSS treatment does not alter sequence composition. (**A**) Left panel: Sequence composition of the CymRSV libraries. The top 10% of the most highly expressed reads were excluded so that highly expressed individual species did not dominate the analysis. Right panel: sequence composition of the CymRSV genome on the minus strand, plus strand or total (‘both’). (**B**) Sequence composition of non-redundant *A. thaliana* small RNA species in either ‘UP’ (over-represented in FDFSS libraries - differentially expressed with a likelihood of >0.9), ‘DOWN’ (over-represented in NSS libraries – differentially expressed with a likelihood of >0.9), ‘NDE’ (non-differentially expressed with likelihood of >0.9) or ‘ALL’ (all small RNA species) at every position (5′-3′) along the length of 21nt (left panel) or 24nt (right panel) small RNAs. ‘n’ indicates the number of small RNA species in each category. (**C**) Sequence composition of *A. thaliana* miRNAs that are present in either FDFSS or NSS libraries (non-redundant reads) (created by WebLogo). (**D**) Sequence composition of the 5′ end of redundant *A. thaliana* miRNA mapping reads in FDFSS or NSS treatments with (left panel) or without miR166/165 (right panel). (**E**) FDFSS does not bias towards/against particular nucleotides: FDFSS/NSS fold change for *A. thaliana* small RNA species (y-axis) against the proportion of a particular nucleotide within the species (x-axis) (C – upper left, A – upper right, G – lower left or U – lower right). Red line indicates local regression fit (loess).

To find out whether FDFSS treatment introduces a sequence composition bias in sRNA data (31) we first examined the CymRSV mapping reads. We compared the total nucleotide composition of the small RNAs under the three treatments. We excluded the top 10% most highly expressed reads so that that a small number of highly abundant individual species would not dominate the analysis and we observed a very modest difference in sequence composition between the treatments, with FDFSS having more Cs than NSS and with FSS being intermediate (see Figure 4A). However, this difference can be accounted for by the release of vsRNAs derived from the C-rich minus strand (28% versus 23% for the plus strand) by the FDFSS treatment.

Next, we determined whether the *A. thaliana* libraries indicated a sequence bias due to the FDFSS treatment. Using baySeq (51) we separated the small RNAs that were: over-represented in FDFSS (up; differentially expressed with a likelihood of >0.9); under-represented in FDFSS (down; differentially expressed with a likelihood of >0.9); non-differentially expressed (NDE; with a likelihood of >0.9 being NDE). We also considered all small RNA species (all). We then plotted the sequence composition at every position for the small RNA species (non-redundant reads) in these four categories. We hypothesised that, if the FDFSS treatment favored a particular nucleotide sequence, then we would see a difference in the base composition of small RNAs that constitute the Up versus the NDE and/or ALL categories. However, we did not see any significant difference between the categories (see Figure 4B). In particular, the ‘Up’ category strongly resembled the ‘All’ category, suggesting that the ‘Up’ sRNAs are a representative subset of ‘All’. The greatest amount of variation in sequence composition was along the length of the small RNA, consistent with AGO binding preferences having a predominant role in determining sequence composition (44,52–53). It is likely that AGO selection accounts for the 5′ U preference in miRNA reads (54,55) and the A preference for 24nt heterochromatic siRNAs in both FDFSS and NSS libraries (Figure 4B, C and D).

Finally, we were interested to determine whether the proportion of particular nucleotides might be associated with changes in FDFSS. Formaldehyde may modify C and A nucleotides by mono-methylol addition (56) and thereby affect cloning or PCR amplification efficiency during library generation. However, we saw only minor effects on FDFSS enrichment in opposing directions as C and A compositions increased (Figure 4E). Therefore, it seems unlikely that formaldehyde-induced nucleotide modifications are a driving force behind small RNA representation. Taken together, these results confirm that FDFSS treatment does not significantly alter the nucleotide sequence composition of the small RNA pool and indicate that individual differences in small RNA abundance associated with this method are likely to be driven by the effects of sequestration.

## CONCLUSION

For the majority of small RNAs species, methods for detection are generally accurate and consistent between different techniques. However, we show here that when excess complementary RNAs are present, such as during viral infection or when miRNAs are subjected to control by ceRNAs, detection of cognate small RNAs can become distorted. In light of these findings, the interpretation of some reported changes in miRNA/sRNA expression may have to be revised, particularly in cases of anti-correlation between miRNA levels with target transcripts.

To circumvent the potential for sRNA sequestration by complementary RNA, we have developed a novel formaldehyde-based denaturing method of sRNA separation (FDF-PAGE) that releases the full suite of sRNAs from any complementary RNAs in the sample. This technique eliminates a bias in vsRNA distribution and it may be useful to identify lowly expressed or cell type specific sRNAs as it effectively uncouples sRNA representation from the abundance of complementary transcripts. Comparison of FDF-PAGE to non-denaturing methods is a good first step in the identification of sRNAs under the control of ceRNAs.

## SUPPLEMENTARY DATA

Supplementary Data are available at NAR Online.

SUPPLEMENTARY DATA
