# Supplementary material for: FDF-PAGE: a powerful technique revealing previously undetected small RNAs sequestered by complementary transcripts
Source: Nucleic Acids Res. 2015 Jun 13;43(15):7590–9. doi: 10.1093/nar/gkv604 (PMC4551911; doi:10.1093/nar/gkv604)
Supplement: SUPPLEMENTARY DATA [file supp_43_15_7590__index.html]

FDF-PAGE: a powerful technique revealing previously undetected small RNAs sequestered by complementary transcripts — SUPPLEMENTARY DATA 

# FDF-PAGE: a powerful technique revealing previously undetected small RNAs sequestered by complementary transcripts

## SUPPLEMENTARY DATA

- SUPPLEMENTARY DATA
- SUPPLEMENTARY DATA
- SUPPLEMENTARY DATA
- SUPPLEMENTARY DATA
