# Supplementary material for: FDF-PAGE: a powerful technique revealing previously undetected small RNAs sequestered by complementary transcripts
Source: Nucleic Acids Res. 2015 Jun 13;43(15):7590–9. doi: 10.1093/nar/gkv604 (PMC4551911; doi:10.1093/nar/gkv604)
Supplement: SUPPLEMENTARY DATA [file supp_gkv604_nar-03730-y-2014-File007.pdf]

Supplementary Materials for

FDF-PAGE: A Powerful Technique Revealing Previously Undetected Small RNAs Sequestered by Complementary Transcripts

C. Jake Harris[1], Attila Molnar[1,2], Sebastian Müller[1], David Baulcombe[1*]

[1] Plant Sciences Department, Cambridge University, UK

[2] School of Biological Sciences, Edinburgh University, UK

* To whom correspondence should be addressed. Tel: +44 1223 333900

Email: dcb40@cam.ac.uk

Supplemental Figure 1: Fully Denaturing Formaldehyde (FDF) treatment relieves sRNA sequestration

A) Northern blot with 21_S (2nM) mixed with x10, x100 or x1000 concentration of a 40nt RNA oligo with antisense complementarity to 21_S (40_AS) or a randomly version of this RNA oligo (40_shuff) under standard RNA pre-loading F treatment **B)** Northern blot for 21_S (2nM) mixed with x10 or x100 molar excess 21_AS under (F) treatment (left half) or boiling in 100% formamide (right half). 21_S (2nM) is also mixed with water as a negative control or a non-complementary 21nt RNA oligo (21_NC) at x100 molar excess as sequence specificity control. **C)** Northern blot after 21_S (2nM) is mixed with x100 21_AS under F, FDF, Glyoxal/DMSO or 8.5M urea pre-loading treatments.

Supplemental Figure 2: The presence of complementary RNA reduces small RNA cloning efficiency

A) Composition of RNA used to make two sRNA libraries. Total RNA from *Arabidopsis thaliana* (1ug) was spiked with 6fmol 21_S oligo + 6pmol (x1000) 40_AS or 6pmol (x1000) 40_shuff oligos **B)** Schematic of primer design to specifically amplify sRNA of interest after using the Illumina sRNA cloning protocol for adapter ligation and cDNA synthesis. **C)** qPCR of the cloned 21_S sRNA normalised to three different endogenous Arabidopsis sRNAs (miR166, AtRep2 and miR173). Fold change between libraries 1 and 2 are indicated. Error bars represent 95% confidence intervals.

Supplemental Figure 3: CymRSV infected *N. benthamiana* small RNA libraries

A) Table summarizing the small RNA libraries generated from CymRSV infected *N. benthamiana* using NSS, FSS and FDFSS treatments **B)** size class distributions of CymRSV mapping vsRNAs **C)** Hierarchical clustering of CymRSV mapping vsRNAs libraries (darker indicates increased similarity). **D)** Distribution of vsRNAs along the length of the CymRSV genome (as in Fig. 2) compared to datasets from Szyttya et al., 2010 sequenced with Illumina, (for the Szyttya vsRNA plot, red, green, yellow, blue represent 21, 22, 23, 24nt small RNAs, respectively). **E)** Model for the cause of vsRNA strand bias under

non-fully denaturing conditions: Upon entering the cell, positive sense RNA viruses (blue) replicate their genomes from a negative stranded template (red) generated by the action of viral replicases/host RDRs. These long double-stranded RNAs are targeted by DCL enzymes for cleavage into vsRNAs. The (-) vsRNAs are sequestered by the highly abundant (+) viral genome resulting in a depleted pool of (-) vsRNAs, creating on observed (+) vsRNA sense strand bias. FDF-PAGE releases all small RNAs from sequestration so that the full pool of small RNAs is available for cloning and sequencing.

Supplemental Figure 4: Endogenous *N. benthamiana* mapping reads in CymRSV infected samples

The same small RNA libraries from CymRSV infected *N. benthamiana* samples were re-mapped against the *N. benthamiana* genome. **A)** size class distribution of *N. benthamiana* aligned reads **B)** Proportion of mature miRNA mapping reads per library under FDFSS, FSS or NSS treatments.

Supplemental Figure 5: Endogenous small RNA library comparisons

A) Table summarizing the small RNA libraries generated from *A. thaliana* (floral, two replicates), *M. musculus* (testis, 25dpp), *D. melanogaster* (whole) and *C. elegans* (whole) libraries under FDFSS or NSS treatment. **B)** Size class distributions for normalized mapped small RNA reads in **A)**

Supplemental Figure 6: Effect of masking miR166/165 from the *A. thaliana* libraries

A) Size class distributions of *A. thaliana* (floral, two replicates) showing all small RNAs (left panel) or with miR166 and miR165 counts masked from the data (right panel). **B)** Proportion of sRNAs mapping to different genome annotation features showing all sRNAs (left panel) or with miR166 and miR165 counts masked from the data (right panel).

Supplemental Figure 7: Top 5 most highly sequestered miRNAs and corresponding miRNA* representations in FDFSS vs. NSS

Tables showing the top 5 most highly sequestered miRNAs from each organism (largest fold change, excluding miRNAs with less than 20 counts in

the NSS libraries) and the representation of their corresponding miRNA* strands. Only small RNA species with >5 reads (cumulative total across any of the libraries) were included. Results show that miRNAs released from the long RNA pool do not cause a consistent reduction in their corresponding miRNA* strand as half of the miRNA*s are also increased in FDFSS.

Oligonucleotide list - all listed 5' to 3' direction:

RNA oligos:

21_S	UUUCAAGGAGGACGGAAACAU
21_AS	GUUUCCGUCCUCCUUGAAAUC
40nt_Shuff	UUAAAUUCCUACUGUCUUCUGUCGUAAGCUCUCCAGCGCC (∆G = -1.9)
40nt_AS	GUUUCCGUCCUCCUUGAAAUCGAUUCCCUUAAGCUCGAUC (∆G = -1.9)
21_NC	CGUGCUGAAGUCAAGUUUGAG

qPCR primers:

F 21_S	ACAGTCCGACGATCTTTCAAGG
R 21_S	AAGACGGCATACGAGATATGTT
F miR166	ACAGTCCGACGATCTCGGACCA
R miR166	AAGACGGCATACGAGATGGGGA
F miR173	ACAGTCCGACGATCTTCGCTTG
R miR173	AAGACGGCATACGAGATGTGAT
F AtRep2	ACAGTCCGACGATCAACGTCCT
R AtRep2	AAGACGGCATACGAGATGCGGG

Probes:

21_S detector	ATGTTTCCGTCCTCCTTGAAA
21_AS detector	GATTTCAAGGAGGACGGAAAC
Probe A	CGAGAAGGAAGCGACGGATTGTT (minus strand, pos 683-703)
Probe B	GGTGCTAGAGGTTCGCACAGGA (minus strand, pos 1129-1150)
Probe C	AGATATCTCAGATACGACGGGGCA (minus strand, pos 4061-4081)
Probe D	AGTCTAACGAAAGTTGAGCTTG (minus strand, pos 4527-4547)
Probe E	GTGCCTGGCAGAATTCAA (plus strand, pos 2160-2180)
Probe F	GGTGCAAAGGGCTACAACCTT (minus strand 3555-3575)
Probe G	GGTACCGTCCTATTAACACCGA (plus strand, 222-243)
Probe H	AACAATCCGTCGCTTCCTTCTCGT (plus strand, 681-701)
Probe I	GGTTTCCACAAATGTTCTCCG (plus strand, pos 4621-

	4641)
Probe J	AGGGAACAAGCTGACAGTGAA (minus strand, pos 3875-3895)
Probe K	GATATCGACGTCTAAGGGTGA (minus strand, pos 1519-1539)
Probe L	GATAGACGAGGTATTGGGATAT (plus strand, pos 516-537)
Probe M	AGCTCCAATATCCCAATACCT (minus strand, pos 509-529)

Supplemental Figure 1: Fully Denaturing Formaldehyde (FDF) treatment relieves sequestration

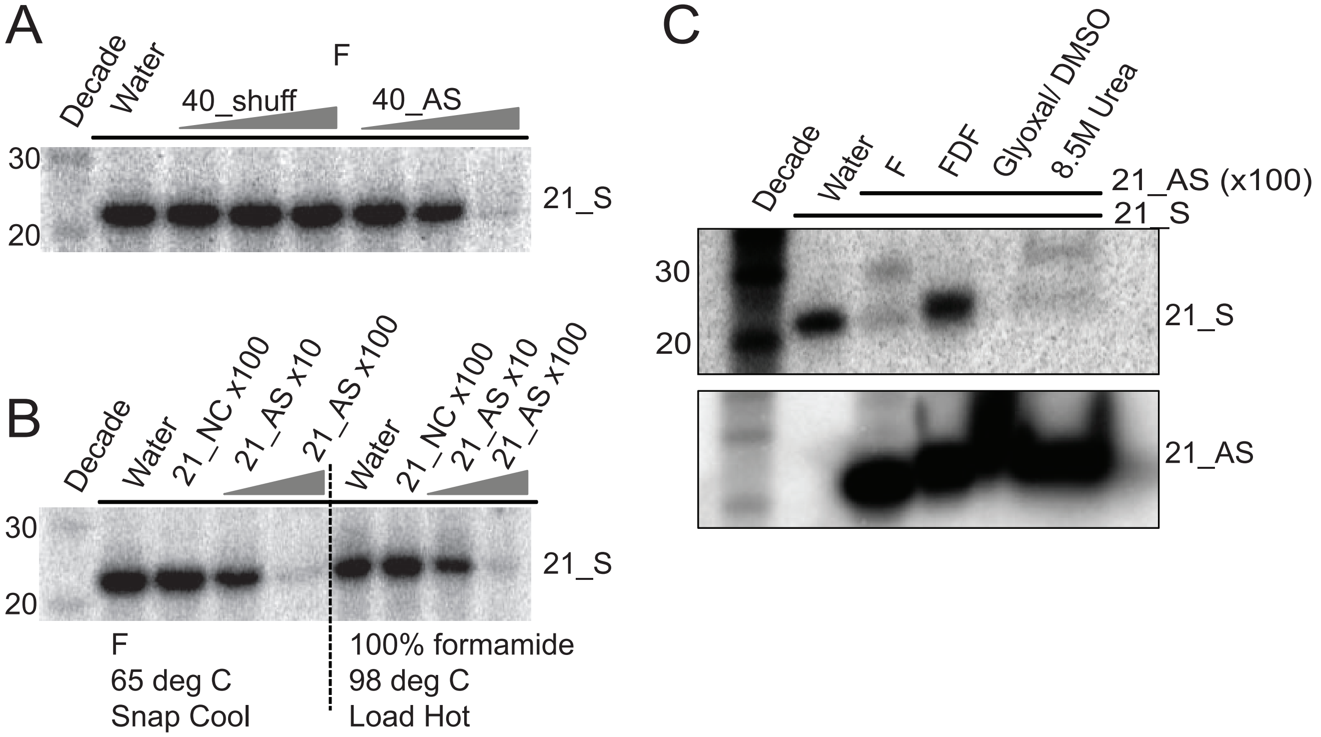

Supplemental Figure 2: The presence of complementary RNA reduces small RNA cloning efficiency

A

Library 1 = Total RNA (1ug) + 21_S (6fmol) + 1000X 40_AS (6pmol)
Library 2 = Total RNA (1ug) + 21_S (6fmol) + 1000X 40_Shuff (6pmol)

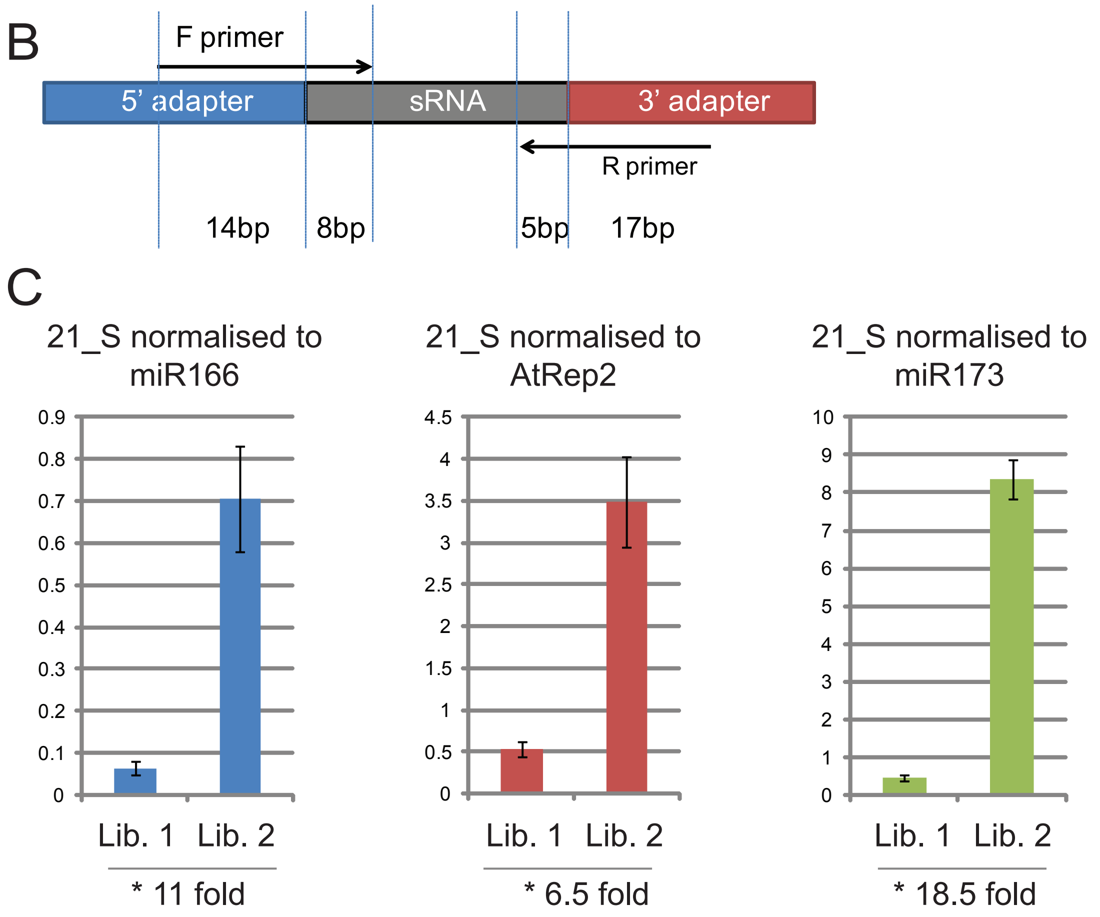

Supplemental Figure 3: CymRSV infected *N. benthamiana* small RNA libraries

A

	raw reads	post/trim filter	%	post align	%	plus	%	minus	%
NSS CymRSV 1	23429748	13269411	56.6	10595757	79.9	9866453	93.1	729304	6.9
NSS CymRSV 2	15316873	9958326	65.0	8331346	83.7	7681074	92.2	650272	7.8
NSS CymRSV 3	18774460	10863195	57.9	8953250	82.4	8329126	93.0	624124	7.0
FSS CymRSV 1	11639868	10655255	91.5	8186470	76.8	5685740	69.5	2500730	30.5
FSS CymRSV 2	10081597	9310384	92.4	7172835	77.0	4777220	66.6	2395615	33.4
FSS CymRSV 3	11572121	10665228	92.2	8309048	77.9	5256320	63.3	3052728	36.7
FDFSS CymRSV 1	10033963	7806977	77.8	6093904	78.1	3230610	53.0	2863294	47.0
FDFSS CymRSV 2	12597907	10332988	82.0	8121467	78.6	4121443	50.7	4000024	49.3
FDFSS CymRSV 3	11257085	9007018	80.0	7092851	78.7	3617214	51.0	3475637	49.0

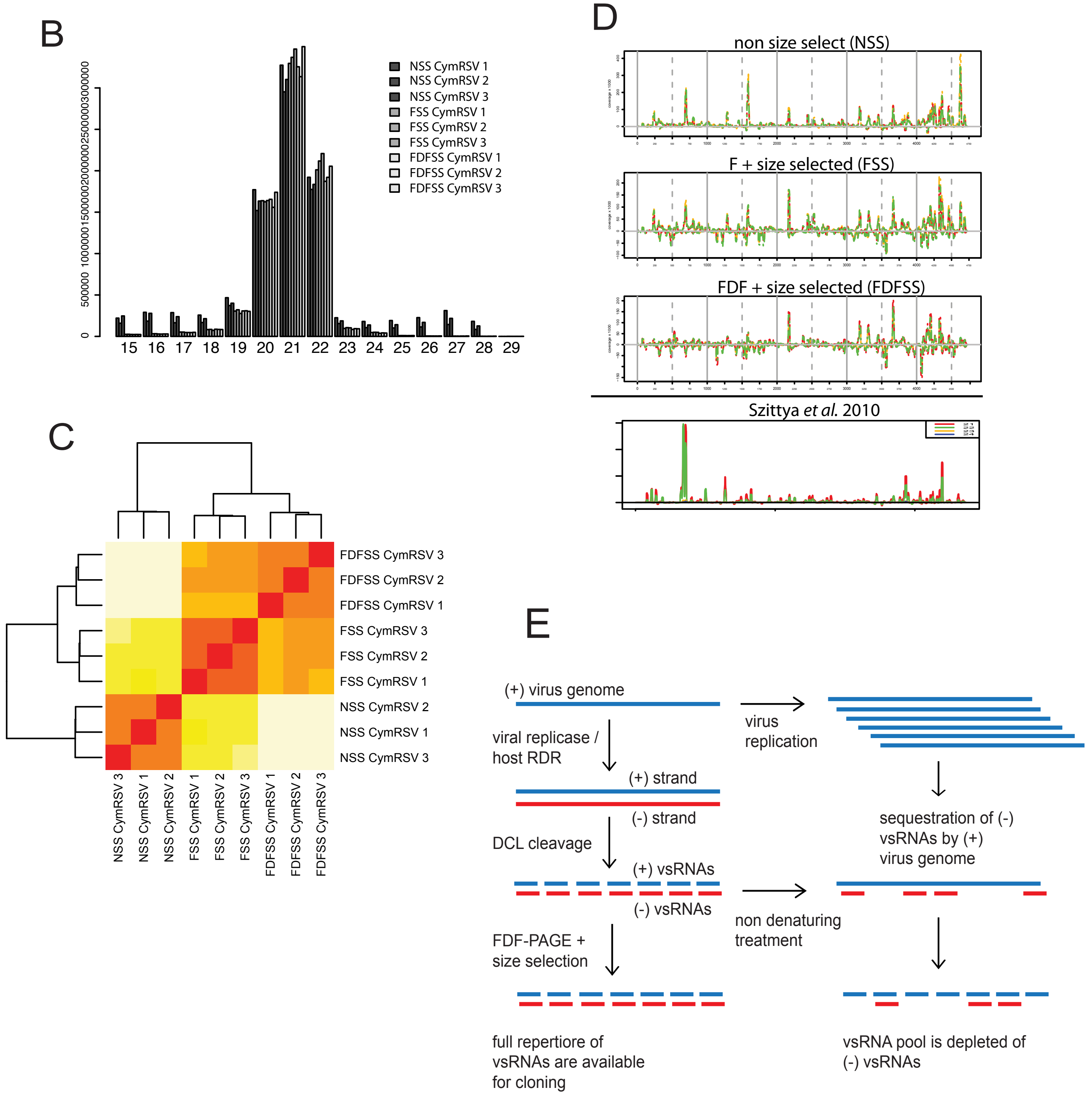

Supplemental Figure 4: Endogenous *N. benthamiana* mapping reads in CymRSV infected samples

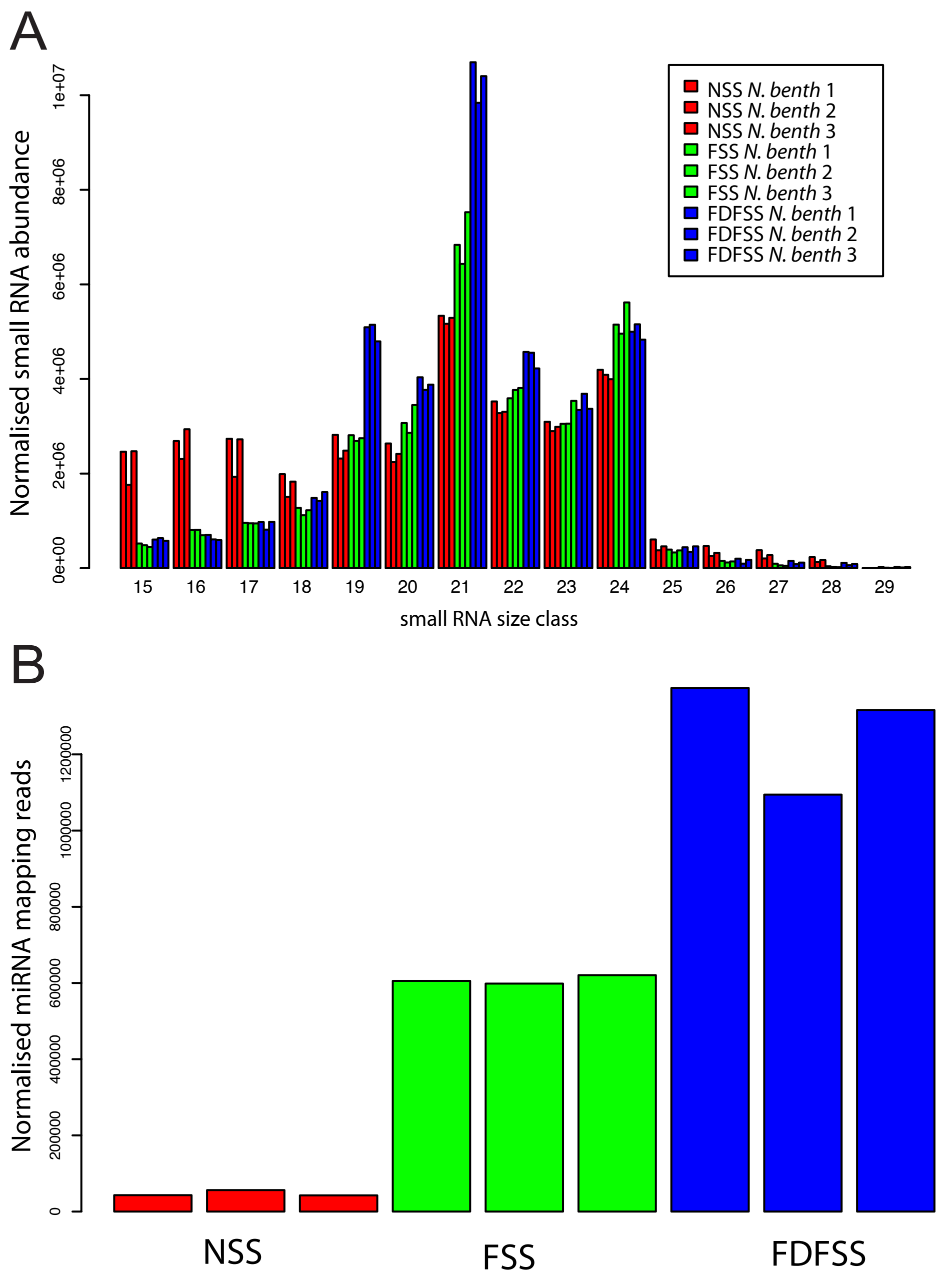

Supplemental Figure 5: Endogenous small RNA library comparisons

A

Library	Raw reads	Post trim/filter	Percentage	After alignment	Percentage
**At1 FDFSS**	7,413,419	5,566,895	75.09	4,646,734	83.47
**At1 NSS**	4,589,772	2,348,983	51.18	2,156,530	91.81
**At2 FDFSS**	6,680,113	5,257,419	78.7	4,539,654	86.35
**At2 NSS**	2,539,611	1,630,516	64.2	1,492,730	91.55
**Mm FDFSS**	35,308,643	12,221,939	34.61	8,884,666	72.69
**Mm NSs**	8,013,553	2,597,928	32.42	2,324,312	89.47
**Dm FDFSS**	11,045,452	4,941,654	44.74	3,457,768	69.97
**Dm NSS**	13,944,461	1,383,024	9.92	1,181,031	85.39
**Ce FDFSS**	11,316,229	6,882,423	60.8	5,853,806	85.1
**Ce NSS**	21,611,776	17,829,058	82.5	16,651,808	93.4

B

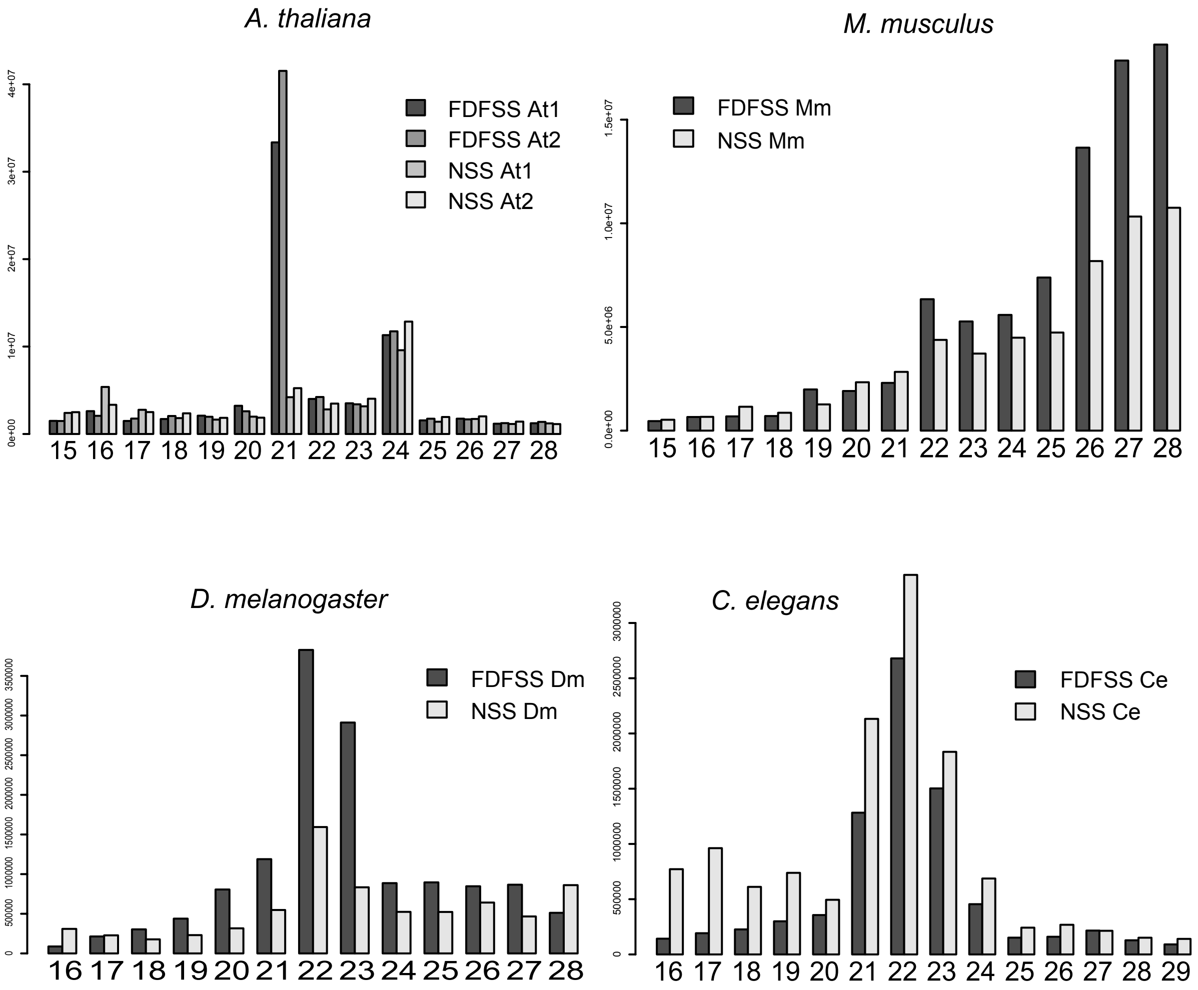

Supplemental Figure 6: Effect of masking miR166/165 from the *A. thaliana* libraries

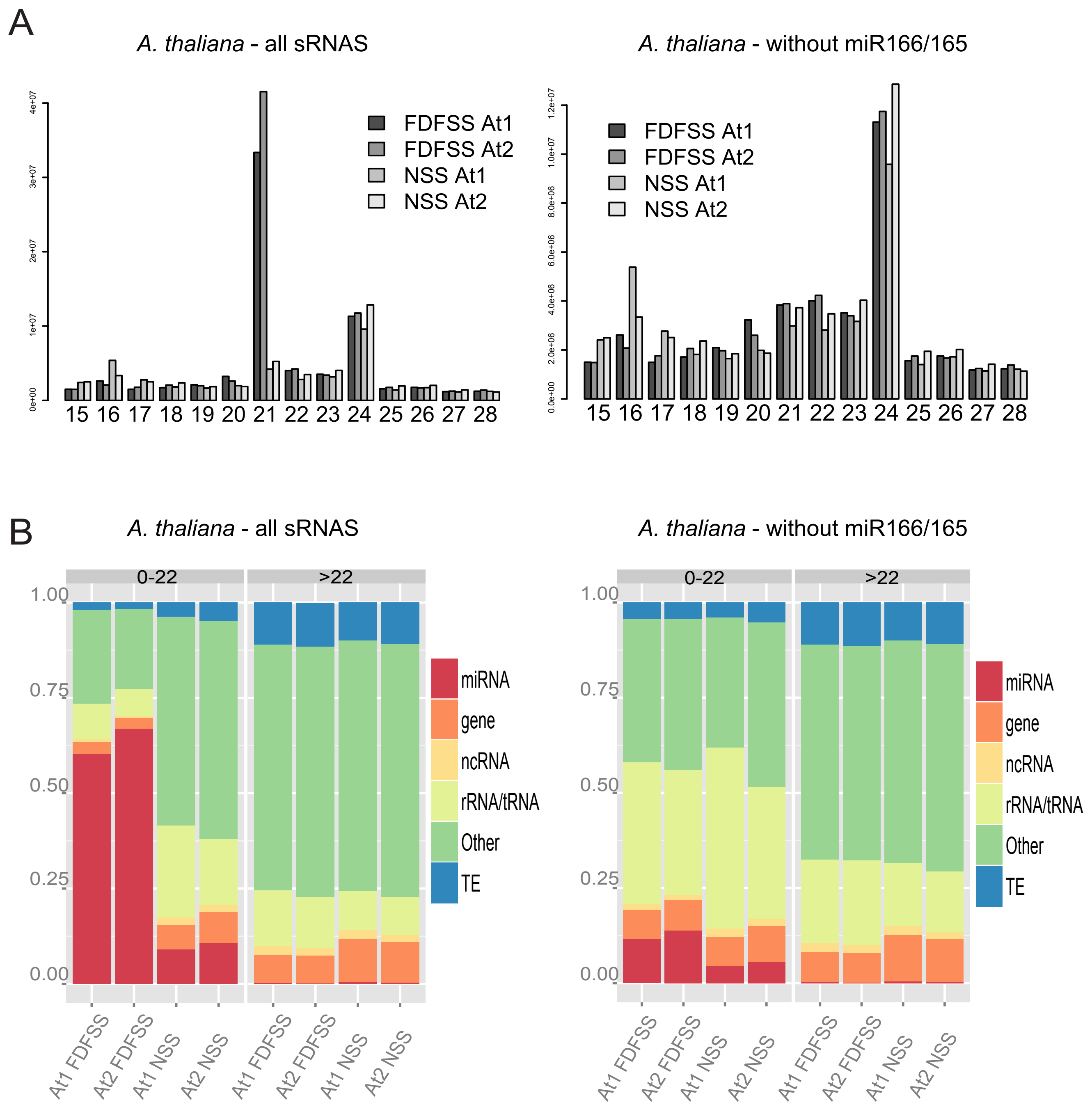

Supplemental Figure 7: Top 5 most highly sequestered miRNAs and corresponding miRNA star representations in FDFSS vs. NSS

A. thaliana		**Normalised reads**			**miRNA Star**	**Normalised reads**		
**miRNA**	**Sequence (5'-3')**	**Avg. FDFSS**	**Avg. NSS**	**Fold Change**	**Sequence (5'-3')**	**Avg. FDFSS**	**Avg. NSS**	**Log_2 ratio (FDFSS/NSS)**
miR166a	TCGGACCAGGCTTCATTCCCC	1298573.8	27232.4	47.7	GGACTGTTGTCTGGCTCGAGG	1085	1526.8	-0.5
miR165a	TCGGACCAGGCTTCATCCCCC	445314.2	15167.6	29.4	GGAATGTTGTCTGGATCGAGG	195.6	72.7	1.4
miR162a	TCGATAAACCTCTGCATCCAG	853.5	68.7	12.4	TGGAGGCAGCGGTTCATCGATC	2.9	6.4	-1.2
miR158a	TCCCAAATGTAGACAAAGCA	18068.1	1571.7	11.5	CTTTGTCTACAATTTTGGAAA	6.9	13.2	-0.9
miR403	TTAGATTCACGCACAAACTCG	966.4	146.5	6.6	TGTTTTGTGCTTGAATCTAATT	14.3	31.7	-1.1

M. musculus		**Normalised reads**			**miRNA Star**	**Normalised reads**		
**miRNA**	**Sequence (5'-3')**	**FDFSS**	**NSS**	**Fold Change**	**Sequence (5'-3')**	**FDFSS**	**NSS**	**Log_2 ratio (FDFSS/NSS)**
miR-92a-3p	TATTGCACTTGTCCCGGCCTG	3869	53.6	72.1	AGGTTGGGATTTGTCGCAATGCT	134	10.7	3.6
miR-125a-5p	TCCCTGAGACCCTTTAACCTGTGA	759.4	21.5	35.4	ACAGGTGAGGTTCTTGGGAGCC	Not present	Not present	
miR-22-3p	AAGCTGCCAGTTGAAGAACTGT	3998.1	157.3	25.4	AGTTCTTCAGTGGCAAGCTTTA	2.8	12.5	-2.2
miR-92b-3p	TATTGCACTCGTCCCGGCCTCC	606.7	25	24.2	AGGGACGGGACGTGGTGCAGTGTT	Not present	Not present	
miR-181a-5p	AACATTCAACGCTGTCGGTGAGT	2158.7	121.6	17.8	ACCATCGACCGTTGATTGTACC	41	1.8	4.5

D. melanogaster		**Normalised reads**			**miRNA Star**	**Normalised reads**		
**miRNA**	**Sequence (5'-3')**	**FDFSS**	**NSS**	**Fold Change**	**Sequence (5'-3')**	**FDFSS**	**NSS**	**Log_2 ratio (FDFSS/NSS)**
miR-996-3p	TGACTAGATTTCATGCTCGTCT	2533.9	98.1	25.8	GCGAACATGGATCTAGTGCACG	31.9	15.2	1.1
miR-133-3p	TTGGTCCCCTTCAACCAGCTGT	4416.3	201.3	21.9	AGCTGGTTGACATCGGGTCAGAT	Not present	Not present	
miR-314-3p	TATTCGAGCCAATAAGTTCGG	896.5	49.1	18.3	GTAACTTGTGTGGCTTCGAACTTACC	149.1	20.3	2.9
miR-286-3p	TGACTAGACCGAACACTCGTGCT	1298.2	74.4	17.4	GGCGAATGTCGGTATGGTCTCT	3.5	1.7	1.1
miR-956-3p	TTTCGAGACCACTCTAATCCATT	205402.4	17873	11.5	GTGTTTGGAATGGTCTCGTTAGCT	58.2	45.7	0.3

C. elegans		**Normalised reads**			**miRNA Star**	**Normalised reads**		
**miRNA**	**Sequence (5'-3')**	**FDFSS**	**NSS**	**Fold Change**	**Sequence (5'-3')**	**FDFSS**	**NSS**	**Log_2 ratio (FDFSS/NSS)**
miR-250-5p	CCTTCAGTTGCCTCGTGATCCG	408.2	42	9.7	AATCACAGTCAACTGTTGGC	796.8	167.8	2.2
miR-90-3p	TGATATGTTGTTTGAATGCCCCT	501057.2	51855.9	9.7	CGGCTTTCAACGACGATATCAAC	18.5	38.1	-1
miR-34-3p	ACGGCTACCTTCACTGCCACCC	5386	862.5	6.2	AGGCAGTGTGGTTAGCTGGTTG	260.5	5227	-4.3
miR-5592-5p	CGGCCCTTACCGTTTAATACATG	136.4	22.4	6.1	TGTATTAAACGGTAAGGGCCGGC	134.3	469.3	-1.8
miR-235-3p	TATTGCACTCTCCCCGGCCTGA	143197.4	29446.4	4.9	AGGCCTTGGCTGATTGCAAAATT	Not present	Not present	
