# Supplementary material for: FDF-PAGE: a powerful technique revealing previously undetected small RNAs sequestered by complementary transcripts
Source: Nucleic Acids Res. 2015 Jun 13;43(15):7590–9. doi: 10.1093/nar/gkv604 (PMC4551911; doi:10.1093/nar/gkv604)
Supplement: SUPPLEMENTARY DATA [file supp_gkv604_nar-03730-y-2014-File008.pdf]

Small RNA separation by Fully Denaturing Formaldehyde Polyacrylamide Gel Electrophoresis (FDF-PAGE) Protocol:

Harris & Molnar *et al.,* 2015

Summary: This protocol describes the separation of small RNAs from total RNA on a polyacrylamide gel. It is based on the typical 7M urea 15% polyacrylamide gel electrophoresis ('denaturing' PAGE) but includes a highly denaturing formaldehyde based incubation step prior to RNA loading. After the electrophoresis is complete, the user can proceed directly to small RNA northern (membrane transfer stage) or can extract the small RNA from the gel and use it as starting material for small RNA library generation.

1) Prepare the gel:

For 15% denaturing polyacrylamide gel (10ml for two 0.75mm thick gels).
Using a 50ml glass beaker, mix the following:

4.2g Urea
0.5ml 10X MOPS
3.75ml 40% (w/v) 19:1 acrylamide:bis-acrylamide
2.5ml water
    stir until the urea is dissolved. Then add:
70µl 10% (w/v) ammonium persulphate (APS)
3.5µl TEMED
    Pour immediately. Polymerisation time is 30 minutes

Assemble electrophoresis equipment. Rinse wells with running buffer (0.5X MOPS), and pre-run the gel at 100V for 30 mins.

2) Prepare RNA samples:
RNA pre-loading treatment:

Prepare in the fume hood. For each gel lane add

V RNA
2.75µl Formaldehyde (40%)
7.5µl Formamide (de-ionised)
0.75µl 10X MOPS
4-V µl water (15ul volume total)

Mix, incubate at 55°C for 15 mins
Add 2µl of 10X Dyes, mix sample, rinse wells then load immediately into gel.

3) Run the gel:

Run the gel at 50V until both dyes enter the gel then increase the voltage up to 150V until Bromophenol blue band reaches the bottom of the gel.

For small RNA Northern protocols: *Proceed with the membrane transfer. See Step 9 from section 3.2. ('Separation of RNA Samples by Denaturing 15% PAGE') of López-Gomollón, 2011 (using 0.5X MOPS instead of 1x running buffer). We recommend 3.3.1 capillary blotting for transfer and 3.4.1 UV crosslinking for crosslink.*

***For small RNA library generation**: see below*

Small RNA gel band extraction:
1) Prize apart the glass plates and stain the gel for 5 min in 1µl SYBR Gold (Life Technologies) per 10ml 0.5X MOPS running buffer (eg. 25ml total).

2) Visualise RNA on a UV or Blue-Light transilluminator. Cut out gel slice between 15-30nt range and place into the 0.5ml tube of a pre-prepared gel shredder unit. (NOTE: small RNA band is usually very faint, use marker lanes to estimate).

> *Gel shredder unit: Use a 21-gauge needle to pierce the bottom of a 0.5ml nuclease free microcentrifuge tube 3-4 times. Place the pierced 0.5ml tube inside a 2ml nuclease free microcentrifuge tube.*

3) Centrifuge gel in shredder unit at 10,000xg for 2 minutes.

4) Remove the empty 0.5ml microcentrifuge tube. Add 3 volumes of ice cold 0.3M NaCl pH 7 per volume of shredded gel in the 2ml tube. **Rotate overnight at 4°C.**

5) Transfer the gel + 0.3M NaCl mixture into a Spin-X column (Costar) by pipetting using a 1 ml tip (cut 3mm off) and centrifuge at 17,000xg for 2 minutes at 4°C

6) Add an additional 100µl of ice cold 0.3M NaCl to the top of the gel in the Spin-X column

7) Centrifuge at 17,000xg for 2 minutes at 4°C

Precipitate the small RNA:
8) Transfer flow through solution into a siliconized low-retention nuclease-free 1.5ml microcentrifuge tube

9) Add 1/10 volume 3M NaOAc pH 5.2 + 1µl of GlycoBlue Coprecipitant (Ambion).

10) Mix with 1 volume of isopropanol per total solution volume (sample + NaOAc + GlycoBlue). Place in -80°C for >30 minutes or -20°C >1h.

11) Centrifuge at >16,000xg for 30 minutes at 4°C.

12) Discard supernatant by pipetting. Make sure that the GlycoBlue-stained pellet remains in the tube. Take care to not disturb the pellet. To wash pellet, add 1ml of ice cold **80% ethanol** and centrifuge at >16,000xg for 5 minutes at 4°C and discard supernatant. Repeat wash. *Note: it is important to use 80% ethanol, as small RNAs are slightly soluble in lower 70-75% ethanol solutions.*

13) Collect the remaining 80% ethanol at the bottom of the tube by a pulse spin. Remove as much ethanol as possible by pipetting and allow pellet to air dry on ice for 5-10 minutes.

14) Re-suspend pellet in 5-10µl of nuclease free water. Can be stored at -80°C **or used directly for small RNA library preparation**. For instance, use 3µl of the

isolated small RNA in step 1 of the ‘Ligate 3’ Adapter’ stage in the TruSeq Small RNA Library Prep Guide and continue with the recommended protocol.

Notes

RNA input recommendations: Total RNA for input can be isolated using a number of methods. For instance, TRIzol (Invitrogen) extraction is recommended. Load the appropriate amount of total RNA for the intended downstream application. For instance, 10-15μg RNA per gel lane is recommended for small RNA northerns (see López-Gomollón, 2011 Note 7) and 1-5μg total RNA is recommended for small RNA libraries (TruSeq Small RNA Library Prep guide, Illumina). However, we recommend using 5-10μg per small RNA library as some small RNA may be lost during gel extraction and precipitation.

Ladders and markers: For small RNA northern, the Decade (Ambion) ladder can be directly added to the gel (cat #AM7778) not requiring the RNA pre-loading treatment described below. For small RNA library generation, the appropriate ladder (eg. Abnova small RNA marker #R0007) should be pre-treated along with the other RNA samples prior to loading.

Useful notes: 1) While formamide treated samples can be run on MOPS or TBE based gels, formaldehyde (FDF) treated samples must be run on MOPS based gels. 2) If NSS vs. FDFSS libraries have been prepared, one might not be able to validate the small RNAs that are increased in FDFSS by northern as this necessarily includes size selection. One can only expect to validate FSS vs. FDFSS library differences by northern.

Buffers and Reagents for FDF-PAGE

10X Dyes:
10ml nuclease free, de-ionised water
5mg xylene cyanol FF
5mg bromophenol blue

Gel running buffer:
0.5X MOPS

10X MOPS Buffer (for 1L):

200mM MOPS pH 7.0	(41.6g MOPS)
50mM NaOAc	(4.1g NaOAc (anhydrous))
10mM EDTA 20ml	(0.5M Na_2EDTA pH8.0)
pH to 7.0 with NaOH	

Store in dark bottle

References:

López-Gomollón, S. (2011). Detecting sRNAs by Northern Blotting. *Methods in Molecular Biology*, *732*(4), 25–38. doi:10.1007/978-1-61779-083-6
